# Glucose and the Injured Brain-Monitored in the Neurointensive Care Unit

**DOI:** 10.3389/fneur.2014.00091

**Published:** 2014-06-06

**Authors:** Elham Rostami

**Affiliations:** ^1^Department of Neuroscience, Section of Neurosurgery, Uppsala University, Uppsala, Sweden; ^2^Department of Neuroscience, Karolinska Institutet, Stockholm, Sweden

**Keywords:** glucose, microdialysis, subarachnoid hemorrhage, Traumatic brain injury, intensive insulin therapy, hyperglycemia, neurocritical care, neuromonitoring

## Abstract

Brain has a continuous demand for energy that is met by oxidative metabolism of oxygen and glucose. This demand is compromised in the injured brain and if the inadequate supply persists it will lead to permanent tissue damage. Zero values of cerebral glucose have been associated with infarction and poor neurological outcome. Furthermore, hyperglycemia is common in patients with neurological insults and associated with poor outcome. Intensive insulin therapy (IIT) to control blood glucose has been suggested and used in neurointensive care with conflicting results. This review covers the studies reporting on monitoring of cerebral glucose with microdialysis in patients with traumatic brain injury (TBI), subarachnoid hemorrhage (SAH) and ischemic stroke. Studies investigating IIT are also discussed. Available data suggest that low cerebral glucose in patients with TBI and SAH provides valuable information on development of secondary ischemia and has been correlated with worse outcome. There is also indication that the location of the catheter is important for correlation between plasma and brain glucose. In conclusion considering catheter location, monitoring of brain glucose in the neurointensive care not only provides information on imminent secondary ischemia it also reveals the effect of peripheral treatment on the injured brain.

## Importance of Glucose Monitoring in Neurointensive Care

The development of neurointensive care (NIC) has had a huge impact on improving outcome and reducing mortality in patients with critical neurological conditions (1–4). This NIC includes mainly care for patients with traumatic brain injury (TBI), subarachnoid hemorrhage (SAH), intracranial hemorrhage, spinal cord injury, and acute ischemic stroke. The acute injured brain is characterized by a primary and a secondary injury. Primary brain injury is the acute insult to the brain that can be ischemia, hemorrhage, or trauma among others and is irreversible.

The different types of primary injuries trigger secondary injury processes such as posttraumatic ischemia, energy failure, excitotoxicity, mitochondrial failure, oxidative stress and release of free radicals, secondary cerebral swelling, and inflammation (5, 6).

Ischemia plays a major role in the pathology of injured brain and low cerebral glucose values are detected in ischemia.

The injured brain might also be subjected to secondary clinical insults, e.g., high intracranial pressure, hypoxia, hyperglycemia, and hypoglycemia. Hyperglycemia is a common secondary insult in TBI, SAH, and acute ischemic stroke and has repeatedly been associated with poor neurological outcome. A great challenge for the treatment of patients with acute brain injury in the NIC unit is to detect early signs of secondary injuries in order to prevent further advancement and deterioration of the brain tissue. Microdialysis is a widely used technique to monitor the metabolic state of the injured brain and detect metabolic crises defined as low glucose and high lactate/pyruvate ratio (7–9). Monitoring of brain glucose has become even more important due to the increasing interest in controlling blood glucose within defined limits.

Two landmark studies showed that tight glucose control in critically ill surgical patients, aiming for blood glucose in the range 4.4–6.1 mmol/l, reduced mortality and morbidity (10, 11). However, these results were later challenged by Finfer et al., who showed an increase in mortality when intensive glucose control was used to treat hyperglycemia (12).

This review will focus on monitoring of cerebral glucose in the most common diagnoses present in the NIC; TBI, ischemic stroke and SAH. It will also cover clinical studies investigating treatment of hyperglycemia in the NIC.

## Glucose and the Brain

Brain has a continuous demand for energy that is met by oxidative metabolism of oxygen and glucose. Inadequate supply of oxygen or glucose causes cognitive dysfunction and dependent on the duration and severity there will be a progressive deterioration from coma to persistent brain damage and eventually death.

Glucose is the main substrate used by the brain under normal conditions, glycogen and high-energy phosphate compounds such as phosphocreatine and adenosine phosphates only support neuronal functions for 1–3 min (13). During recent years the astrocyte–neuron lactate shuttle (ANLS) hypothesis has emerged. This hypothesis states that astrocytes produce lactate, which is then taken up by the adjacent neurons and used as an alternative energy substrate (14).

In the normoxic brain more than 95% of the adenosine triphosphate (ATP) is derived from aerobic glucose oxidation. Each molecule of glucose is oxidized by 6 molecules of oxygen to carbon dioxide and water, yielding 38 molecules of ATP. Under fully aerobic conditions, lactate production accounts for <4% of the glucose metabolized (15). However, the anaerobic glycolysis that breaks down glucose to lactate and pyruvate yields only two molecules of ATP for each molecule of glucose (16). The speed of ATP production is dramatically increased compared to oxidative phosphorylation. During complete ischemia glycolysis is upregulated by seven- to eightfold, within 30 s all the glucose and glycogen are consumed and by 1 min all ATP (17, 18).

During inadequate oxygen supply each ATP molecule generates a hydrogen ion and coupled with lactate production leads to lactic acidosis. The extent of lactic acid production is dependent on the preischemic levels of glucose and glycogen (19). The intracellular acidosis that is produced is deleterious for the neurons, nevertheless, it is not the lactate in itself that is harmful. Rather the intracellular increase in hydrogen ion concentration is believed to be cytotoxic (20). Interestingly, moderate increase in lactate post ischemia has been suggested to have neuroprotective effect (21).

Hyperglycemia or hypercapnia exacerbates ischemic damage, indicating that low pH in combination with ischemia and/or reperfusion enhances detrimental processes and cell death (22–24).

Glucose enters the brain through facilitated diffusion via glucose transporters in the blood–brain barrier (BBB). There is a coupling between BBB glucose transport and cerebral metabolic rate of glucose (25).

Plasma glucose concentrations are normally maintained between 3.0 and 5.6 mM, but can vary between 2 and 10 mM or higher in pathological conditions. Within the brain however, cerebrospinal fluid is buffered to the extent that the range within which glucose concentrations vary is much lower and narrower (0.5–2.5 mM).

## Brain Microdialysis and Glucose

**Figure 1:**
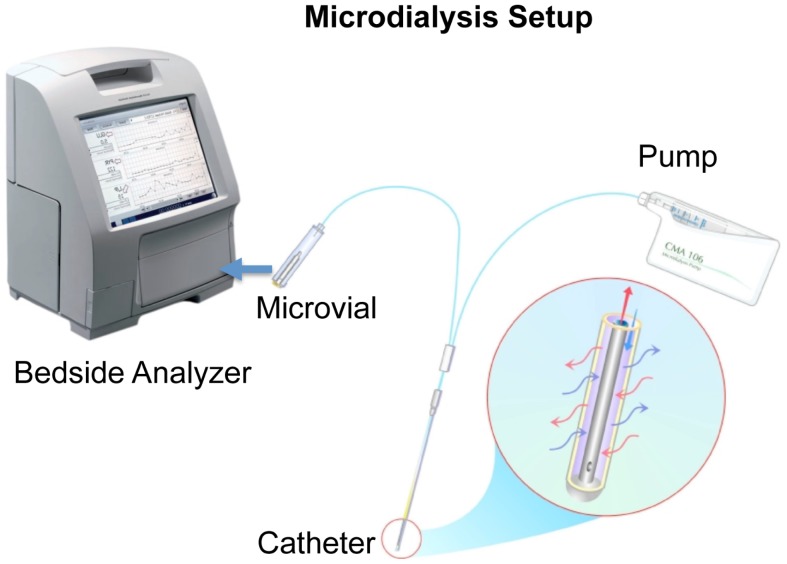
**A schematic picture of microdialysis setup is presented courtesy of M dialysis AB**. The catheter is inserted in the brain tissue and a physiological salt solution is slowly and constantly pumped through a semipermeable membrane. This dialysis membrane at the distal end of the microdialysis catheter functions like a blood capillary. Chemical substances from the extracellular fluid diffuse across the membrane into the perfusion fluid inside the catheter. The “microdialyzate” is then collected in a microvial and analyzed in the bedside analyzer ISCUSflex.

Microdialysis can be used to monitor the metabolic state of almost any tissue and is a widely used technique for monitoring brain energy metabolism during neurointensive care (Figure 1) (8). It was initially used in rodents studying neurotransmitters (26) and later developed to be used in humans to monitor brain metabolic state (27).

A microdialysis catheter forms a “biosensor.” A semipermeable membrane with a double-lumen concentric cannula, mimicking a blood capillary, is attached to the microdialysis catheter. The catheter has an inlet and outlet tube. A sterile fluid is perfused through the inlet tube, and chemical substances from the interstitial fluid diffuse across the membrane into the perfusion fluid in the inner cannula. The inner cannula connects to the outlet tube that ends in a vial holder where the fluid, now referred to as dialyzate, is collected.

The recovery of a substance is defined as the concentration of the substance in the dialyzate expressed as a percentage of the concentration in the interstitial fluid, which is usually assumed to be similar to blood. If the semipermeable membrane is long enough and the perfusion flow slow enough, the concentration in the dialyzate membrane will approach the concentration in the interstitial fluid, i.e., recovery will be close to 100%.

The availability of modern analytical techniques has made microdialysis a biosensor capable of monitoring essentially every small and medium sized molecular compound in the interstitial fluid of endogenous as well as exogenous origin.

Normal brain glucose levels have been measured by microdialysis in patients undergoing surgery to treat benign lesions in posterior fossa (28). The cerebral glucose with a perfusion rate of 0.3 μl/min in anesthetized patients was 1.2 ± 0.6 mmol/l and awake patients 1.7 ± 0.9 mmol/l.

Alteration in dialyzate glucose results from several reasons (Table 1):

– Ischemia caused by insufficient blood flow causing decrease levels of tissue glucose and oxygen.
– Hyperemia due to increased blood flow and thereby increased glucose delivery.
– Hyperglycemia due to increased blood glucose that increases the dialyzate glucose.
– Hyper- or hypometabolism this will cause an increase or decrease of glucose uptake into the cells and thereby affect the extracellular glucose available to the microdialysis catheter.

**Table 1:** **The table presents conditions that can lead to low or high dialyzate glucose**.

High dialyzate glucose	Low dialyzate glucose
*Hyperemia* due to increased blood flow and thereby increased glucose delivery	*Ischemia* caused by insufficient blood flow causing decrease levels of tissue glucose
*Hyperglycemia* due to increased blood glucose that increases the dialyzate glucose	*Hypoglycemia* due to decreased blood glucose that decreases the dialyzate glucose
*Hypometabolism* this will cause a decrease of glucose uptake into the cells and thereby lead to high extracellular glucose available to the microdialysis catheter	*Hypermetabolism* this will cause an increase of glucose uptake into the cells and thereby lead to low extracellular glucose available to the microdialysis catheter

## Ischemic Stroke and Glucose

Extensive research has been performed on ischemic stroke and a review of these is outside the scope of the current paper. Ischemic infarcts are usually not admitted to the NIC unit and thus do not receive invasive neuromonitoring. This is because they have typically motor or sensory deficits with little or no impairment of consciousness. A group of ischemic stroke patient that do attend the NIC unit are patients with massive or malignant infarcts and thus are the patient group in focus in this review.

Massive hemispheric infarctions constitute 10% of hemispheric strokes and 5% of all ischemic strokes and have a mortality rate of 50–80%, which led to the term malignant infarct (29, 30).

Infarcts in these patients are followed by a space occupying brain edema causing malignant midline shift and compression of the basal cisterns on neuroimaging.

Admission hyperglycemia has been shown to be present in more than one-third of patients with acute ischemic stroke and is significantly more common in those with more severe strokes (31).

Several studies both in humans and animals have shown worsening neurological outcome following preischemic hyperglycemia. The first study was performed in monkeys receiving glucose solution before cardiac arrest that exacerbated the neurological and histopathological outcome (22). Several additional studies have repeated these result following global ischemia in monkeys (32), cats (33), and dogs (34, 35) showing increased neuronal cell death, neurological dysfunction, and mortality. In rats, preischemic hyperglycemia induced post injury seizure and increased structural damage (36, 37).

A systemic review and meta-analysis of the middle cerebral artery (MCA) occlusion model showed that the infarct size of the hyperglycemic animals was 94% larger than normoglycemic animals (38). However, the relevance of these animal hyperglycemia models to the clinical conditions was questioned.

Several studies have shown that hyperglycemia in patients with acute stroke is associated with poor outcome (39–45). This was a key-contributing factor in generating glucose treatment with insulin therapy. Several randomized clinical studies have evaluated the effect of intensive insulin therapy (IIT) (46–53), they included small number of patients and with no conclusion on the clinical efficiency of IIT. The largest randomized clinical trial was UK Glucose Insulin in Stroke Trial, which enrolled 933 patients and showed no clinical benefit of IIT (48). However, the study has been criticized for several significant weaknesses that cause interpretation difficulties.

In a recent randomized study INSULINFARCT trial, 180 patients with acute stroke were randomized to receive IIT or subcutaneous insulin treatment during the first 24 h (54). It was shown that IIT in the first 24 h was associated with larger infarct growth and was not recommended.

There are a few studies that have used microdialysis in patients with ischemic stroke but unfortunately none of them report on dialyzate glucose. Dohmen et al. used cerebral microdialysis in patients with MCA infarction to predict malignant course, but dialyzate glucose was not analyzed (55). Additional studies have used cerebral microdialysis in patients with ischemic stroke but did not report on the dialyzate glucose (55–60).

## Subarachnoid Hemorrhage and Glucose

It is estimated that 1–7% of all strokes are SAH (61). SAH has a huge impact because of the relatively young age of onset and high morbidity and mortality. In aneurysmal SAH, 10–15% of the patients die before reaching medical care, more than half of the patients die within 2 weeks and the overall mortality is 45% (62, 63). For the survivors observation and monitoring is necessary in order to prevent and detect possible secondary insults.

Cerebral ischemia is one of the devastating secondary insults in SAH (64). This is sometimes reversible, but may also progress to infarction, which is associated with increased mortality and severe disability (65, 66). Detection of early perturbation of energy metabolism and cerebral ischemia is highly important in NIC management of SAH patients (67).

In many clinics around the world, cerebral microdialysis is used routinely to detect metabolic disturbances in patients with SAH. Monitoring of brain glucose in these patients has shown to provide essential information. Persson et al. showed that in patients who develop an infarct the glucose values in the MD catheter area decrease to zero and zero values of glucose were detected in patients with unfavorable outcome (68).

The association of zero cerebral glucose value and ischemia in SAH patients was also shown by Schulz et al. They observed significantly lower levels of glucose in patients with severe and complete ischemia when compared with patients without symptoms of ischemia (glucose 0 compared with 2.12 ± 0.15 mmol/l) (69). Decreasing levels of brain glucose and increasing lactate/pyruvate ratio have shown to predict new infarcts in the territory of the microdialysis catheter (70). A low level of glucose (≤0.7 mmol/l) and high L/P ratio (≥45) was used to define metabolic crises and these were associated with low cerebral perfusion pressure and worse outcome (71).

Delayed cerebral ischemia caused by vasospasm is a common contributing factor to increased morbidity and mortality in SAH. Many methods have been developed to detect and monitor signs of vasospasm including monitoring ischemic metabolites (72, 73). Microdialysis catheters were placed in the vascular territory most likely to be affected by vasospasm and it was shown that cerebral glucose was significantly lower in SAH patients presenting signs of clinical vasospasm than in asymptomatic patients (74). Extremely low levels of cerebral glucose were also found in SAH patients with acute ischemic neurologic deficits who developed cerebral infarction (75). Low levels of cerebral glucose have also been associated with poor clinical status or neurological deterioration in SAH patients (76).

The correlation between brain MD glucose levels and plasma glucose concentrations has shown to be heterogeneous with positive, negative, and no correlation at all (77). Plasma glucose concentrations play a major role since hyperglycemia in SAH patients is common and is associated with poor clinical outcome (78–84). One study showed that SAH patients with persistent hyperglycemia are seven times more likely to have poor outcome than patients with normoglycemia (85).

A causal relation between hyperglycemia and poor outcome in SAH patients remains elusive, but it has been suggested that hyperglycemia may exert a detrimental effect by increasing secondary complications such as infection, cerebral ischemia and by facilitating the progression from ischemia to irreversible infarction (86).

These results instigated the need of glucose control and insulin therapy in SAH patients. Several studies have reported on IIT in SAH patients (87–92). One of the major findings is that insulin administration *per se* decreases the brain glucose independent of serum glucose levels.

Schlenk et al. inserted a microdialysis catheter into the vascular territory of the aneurysm after clipping and treated blood glucose levels above 140 mg/dl with intravenous insulin. This induced a decrease of cerebral glucose though blood glucose remained normal (93). These results were confirmed in an additional study where hyperglycemia was not related to high cerebral glucose (93). Low cerebral glucose was more frequently observed in symptomatic patients and with unfavorable outcome if combined with hyperglycemia. They concluded that low as well as high levels of brain glucose could occur independently of blood glucose levels in patients with SAH. Also Schmidt et al. reported an association of insulin administration with a relative reduction of interstitial brain glucose concentrations independent of serum glucose levels (94).

The majority of the studies using IIT reported episodes of hypoglycemia. Episodes of hypoglycemia pose additional risks to the brain with compromised metabolism.

Insulin therapy inducing episodes of low glucose (<4.44 mmol/l) was associated with cerebral infarction, vasospasm, and worse functional outcome 3 months following SAH (92).

Despite several reports on use of IIT in SAH patients there is only one randomized trial where 40 patients receive IIT. This study showed no significant improvement in clinical outcome or the incidence of vasospasm (88).

In conclusion, currently there is no evidence that hyperglycemia in SAH patients should be treated with IIT. This treatment is accompanied by an increase in hypoglycemic episodes, which should raise concerns about the safety of this therapy. Monitoring of cerebral glucose with microdialysis in SAH patients have shown to detect secondary ischemia that could reflect development of vasospasm. It has also been correlated with outcome and thus provides valuable information.

## Traumatic Brain Injury and Glucose

Traumatic brain injury is the leading cause of death in young adults in industrialized nations and in the population under 35 years, the death rate is 3.5 times that of cancer and heart disease combined (95). The primary injury initiates metabolic crises, posttraumatic ischemia, and neuronal death (5, 96). In addition, the injured brain might also be subjected to secondary insults, e.g., hypoxia, hypercapnia, hypocapnia, hypotension, hyperglycemia, and hypoglycemia. A great challenge for the treatment of TBI patients in the NIC unit is to detect early signs of secondary injuries in order to prevent further advancement and deterioration of the brain tissue. Brain microdialysis is widely used to detect ischemia and metabolic crises in TBI (8, 97).

Several studies have reported on increased glycolysis in the acute phase of brain injury (98–100) and low dialyzate glucose levels have been associated with poor outcome (101, 102). Hence the importance of adequate glucose supply from systemic circulation to the injured brain. It has been shown that the intracerebral glucose concentration increased significantly during transient episodes of both moderate and pronounced hyperglycemia (103). Increased dialyzate glucose has shown to be associated with high mortality (104). A linear correlation between peripheral glucose and brain glucose was demonstrated in TBI patients (105). However, there were opposing results in a study monitoring both the injured hemisphere and non-injured hemisphere in TBI patients. While the non-injured hemisphere showed a positive correlation with plasma glucose, the injured hemisphere presented a more heterogeneous pattern with no significant correlation to the blood glucose in the first 12 h of NIC unit (106). This emphasizes the importance of microdialysis catheter placement.

Hyperglycemia is frequently observed in patients with TBI and the degree of hyperglycemia observed can be a predictor of outcome (107–111). As previously discussed, hyperglycemia exacerbates ischemic neurological injury and contributes to poor outcome also in TBI patients. Thus, the effect of insulin therapy has also been studied in TBI patients.

Reducing the plasma glucose by insulin therapy has been shown to decrease cerebral glucose and was associated with brain energy crises in TBI patients. As previously mentioned, Oddo et al. defined brain energy crisis as a cerebral microdialysis glucose <0.7 mmol/l with a lactate/pyruvate ratio >40. It was shown that insulin administration was associated with brain energy crises, which in turn correlated with increased mortality (100). Vespa et al. also showed decreased dialyzate glucose upon insulin therapy but did not find an effect on mortality or functional outcome (112). A retrospective study compared clinical outcomes before and after implementation of IIT in 228 TBI patients. Although episodes of hypoglycemia were significantly more common in the IIT group the overall mortality was similar in both groups (113).

A randomized controlled trial of 97 patients with severe TBI compared a regimen of IIT (target blood glucose 4.42–6.63 mmol/l) versus conventional management (target blood glucose 4.42 and 12.15 mmol/l). The only favorable endpoint associated with the use of IIT was a shorter stay in the NIC unit. No significant differences were observed in rates of mortality and poor functional outcome at 6 months. Meanwhile, the incidence of hypoglycemic events was markedly increased among patients treated with IIT (114). This was confirmed in an additional randomized trial with total of 523 patients including 94 TBI patients. IIT was not associated with improved survival and was associated with increased occurrence of hypoglycemia (115).

In conclusion, current clinical trials do not show any benefit of tight glucose control with IIT in TBI patients. On the contrary it might increase the incidence of hypoglycemia, exacerbating brain metabolic crises (116). Available data suggest that high as well as low cerebral glucose measured by microdialysis is associated with high mortality. There is an indication that the placement of the catheter is important for correlation between plasma and brain glucose.

## Concluding Remarks

There is a vast amount of evidence that hyperglycemia is common in patients with TBI, SAH, and ischemic stroke and that it is related to poor outcome. However, no solid evidence exist that tight glycemic control improves outcome in these patients. It might on the contrary lead to hypoglycemic episode with deleterious effect on the injured brain. Monitoring of glucose with microdialysis has proven to predict ischemic infarcts and detect glucose zero values despite normal blood glucose. Zero dialyzate glucose values are associated with poor outcome. These results also emphasize the importance of catheter location to detect and predict brain tissue at risk of developing infarct.

## Conflict of Interest Statement

The author declares that the research was conducted in the absence of any commercial or financial relationships that could be construed as a potential conflict of interest.
